# An Unusual Case of Nonneutropenic Fever Associated With Myelodysplasia and Sweet's Syndrome

**DOI:** 10.7759/cureus.94166

**Published:** 2025-10-08

**Authors:** Priscila Lopez, Enoch J Abbey, Boakye-Yiadom Adomako, Meena Ahluwalia, Karen Simon

**Affiliations:** 1 Department of Internal Medicine and Infectious Disease, Harlem Hospital Center, Columbia University, New York, USA; 2 Department of Internal Medicine, Harlem Hospital Center, Columbia University, New York, USA; 3 Department of Oncology, Harlem Hospital Center, Columbia University, New York, USA

**Keywords:** leukocytoclasia, myelodysplastic syndrome, neutropenic fever, sweet's syndrome, thrombotic thrombocytopenic purpura

## Abstract

Sweet’s syndrome (SS) is a rare paraneoplastic condition characterized by skin lesions, fever, and leukocytosis. It is typically associated with hematological malignancies but is also linked to solid tumors, medications, and inflammatory diseases. The onset of SS can precede or coincide with the discovery of an undiagnosed cancer. Here, we present the case of a 72-year-old woman with neutropenic fever and an incidental nodule in the right breast. Skin biopsy revealed a neutrophilic infiltrate within the deeper dermis and likely subcutis, which is diagnostic for SS. Successful treatment with systemic steroids was administered, while the concurrent diagnosis of myelodysplastic syndrome led to the initiation of decitabine therapy following a bone marrow biopsy.

## Introduction

Sweet’s syndrome (SS) is also known as acute febrile neutrophilic dermatosis, and was first described by Robert Douglas Sweet in 1964. It is a neutrophilic dermatosis with tender skin lesions, including erythematous papules, plaques, pustules, and nodules, commonly appearing on the upper limbs, trunk, and head and neck [1]. SS can be associated with malignancies, infections, systemic inflammatory disorders, and some medications [2]. It is categorized into classical SS, malignancy-associated Sweet’s syndrome (MASS), and drug-induced SS [3].

MASS may occur in patients with solid malignancies of the genitourinary tract, breast, and gastrointestinal tract, or in association with hematologic conditions, including myeloproliferative, lymphoproliferative, and myelodysplastic disorders. More cases have been described in association with myelodysplastic syndromes (MDSs) and acute myeloid leukemia (AML), and, less frequently, other hematologic malignancies or solid tumors [3,4]. Systemic corticosteroids are the mainstay of therapy for SS. For cases that relapse or are refractory to corticosteroids, agents such as dapsone and colchicine can be used [5].

MDSs are clonal disorders of hematopoietic stem cells characterized by cytopenias, varying degrees of dysplasia, and a risk of progression to AML. They are primarily observed in elderly patients, with the median age at diagnosis being 76 years. The clinical presentation of MDS ranges from indolent conditions with minimal symptoms and mild cytopenias to subtypes that are more comparable to AML [6,7].

Awareness and a high index of suspicion for the rare cutaneous paraneoplastic condition, SS, are essential for accurate diagnosis and prompt treatment. We report a case of MDS with concurrent SS as a paraneoplastic skin condition.

## Case presentation

A 72-year-old woman with a past medical history of hypertension, type 2 diabetes mellitus complicated by proliferative retinopathy and macular edema in both eyes, neovascular glaucoma of the left eye, gout, asthma, bilateral cataract status after surgery with replacement of the right eye lens, breast cancer status post lumpectomy, chemotherapy, and radiation, diverticulosis of the sigmoid colon, and hemorrhoids presented to the emergency room with one day of constant right eye pain associated with headache and dizziness. The patient also reported a decreased appetite, subjective weight loss, fatigue, intermittent shortness of breath, and palpitations for the past three weeks. She also reported skin lesions that appeared two weeks before presentation. The patient denied chest pain, abdominal pain, cough, bleeding, and recent infections. Initial vital signs reported were a blood pressure of 126/48 mmHg, pulse of 83 bpm, RR of 17 bpm, temperature of 98.6°F, and oxygen saturation of 99%. Physical examination was significant for conjunctival pallor, right eye pressure of 17 mmHg, left eye pressure of 22 mmHg with no foreign body or discharge, intact extraocular movements, and no nystagmus; skin examination showed ulcerated plaques and nodules on her trunk and legs; and digital rectal examination showed small external hemorrhoids and thickened internal anal mucosal folds, without evidence of bleeding or mucus. Table 1 shows the results of initial laboratory tests.

**Table 1:** Initial lab results showing complete blood count, anemia panel, and basal metabolic panel HCT, hematocrit; HGB, hemoglobin; LDH, lactate dehydrogenase; MCV, mean corpuscular volume; RBC, red blood cell; WBC, white blood cell; PLT, platelets; TIBC, total iron-binding capacity; BUN, blood urea nitrogen; eGFR, estimated glomerular filtration rate

Parameters	Patient values	Reference range (unit)
WBC	6.34	3.80-10.50 (K/uL)
RBC	2.74	3.80-5.20 (M/uL)
HGB	6.0	11.5-15.5 (g/dL)
HCT	21.2	34.5-45.0 (%)
MCV	77.4	80.0-100.0 (fL)
PLT	20	150-400 (K/uL)
Neutrophil	34.0	43.0-77.0 (%)
Iron	44	30-160 (ug/dL)
TIBC	261	220-430 (ug/dL)
Iron saturation	17	14-50 (%)
Transferrin	201	200-360 (mg/dL)
Haptoglobin	<20	34-200 (mg/dL)
Vitamin B12	496	232-1,245 (pg/mL)
Folate	16.1	≥4.7 (ng/mL)
LDH	1,614	135-214 (U/L)
Reticulocyte	1.57	0.50-1.50 (%)
Sodium	137	136-145 (mmol/L)
Potassium	4.8	3.5-5.1 (mmol/L)
BUN	12	7-18 (mg/dL)
Creatinine	1.0	0.7-1.2 (mg/dL)
Calcium	8.6	8.5-10.1 (mg/dL)
eGFR	64	≥60 (mL/minute/1.73 m^2^)

**Figure 1:**
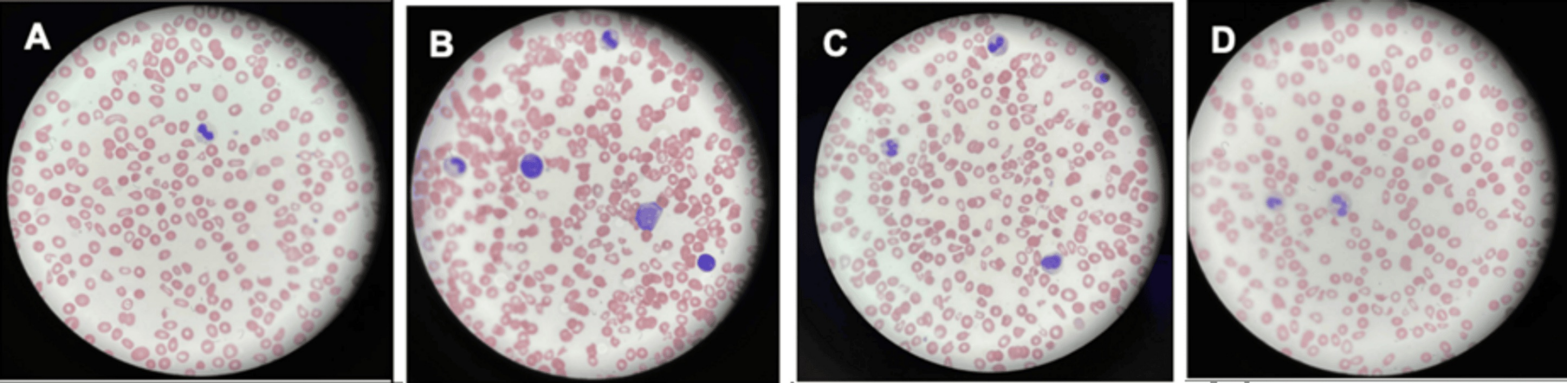
Peripheral blood smear slides showing dysplastic and blast cells, suggestive of evolving MDS. (A) Hypogranular neutrophil. (B) Blast vs. an extended lymphocyte. (C) Nucleated red blood cell at 1 o'clock. (D) Hypogranular and abnormally segmented neutrophil MDS: myelodysplastic syndrome

The patient was admitted for management of severe anemia and thrombocytopenia, as well as new right hyphema and intraocular pressure elevation secondary to neovascular glaucoma. CT of the orbits and head without contrast showed no acute abnormalities. The patient was transfused with two units of red blood cells, while a hematologic workup revealed numerous schistocytes, blast cells, nucleated red cells, hypogranulated neutrophils, and a few thrombocytes (Figure 1). Based on these findings, the hematology consult service recommended a bone marrow biopsy to rule out leukemia and plasmapheresis for suspected thrombotic thrombocytopenic purpura while following up on flow cytometry and ADAMTS13 results. During the third session of plasmapheresis, the patient developed altered mental status, hypertension, tachycardia, and fever. Bloodwork showed a significant drop in the white blood cell count to 1.72 × 10^3^/mcL, and she was initiated on empiric broad-spectrum antibiotics. She was then transferred to a tertiary hospital center for further workup and management.

**Figure 2:**
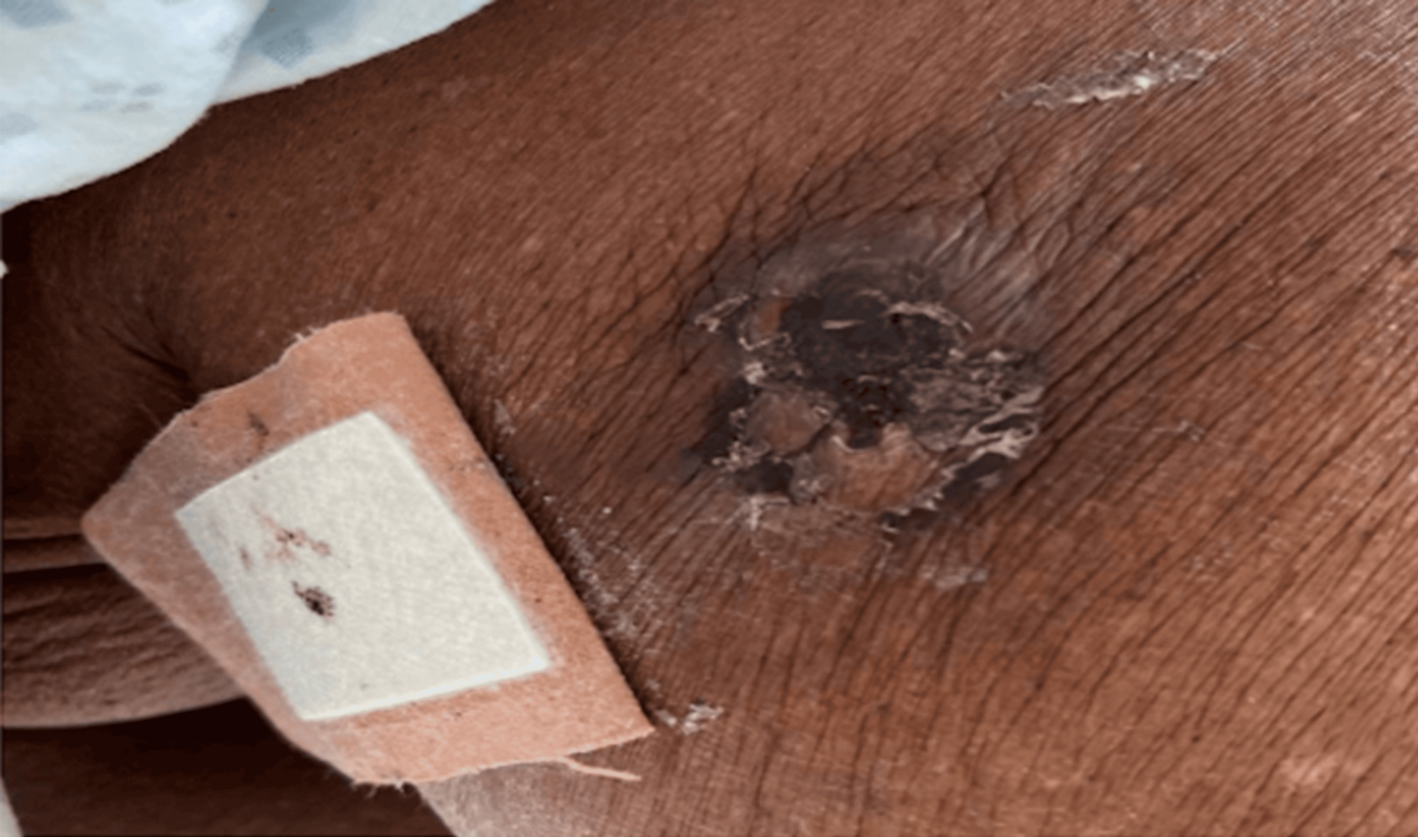
An ulcerated nodule on the patient’s right breast before biopsy

**Figure 3:**
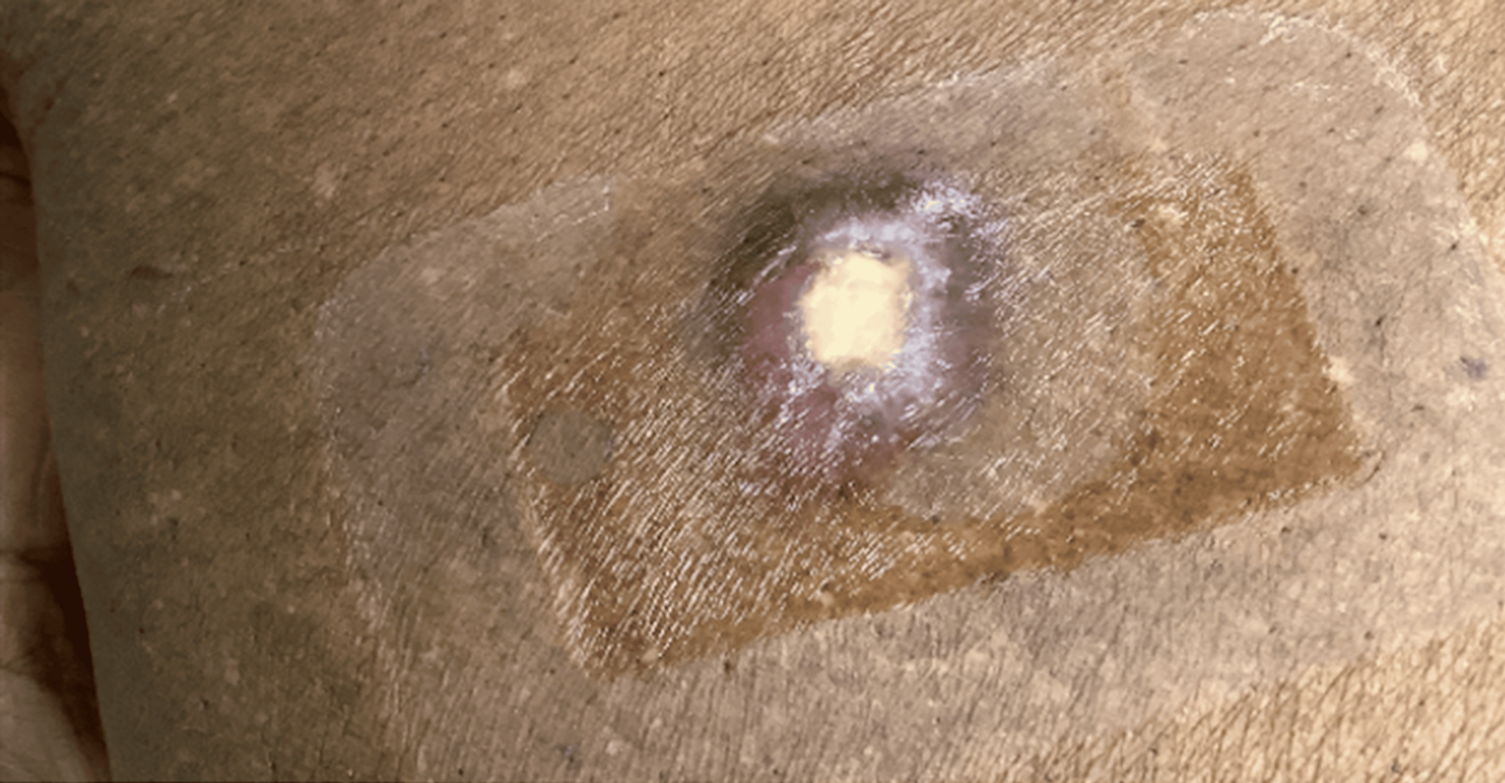
Nodule on the patient's right breast after treatment with steroids

After transfer, the patient underwent a biopsy of a nodule on the right breast (Figure 2). The biopsy revealed an extensive neutrophilic infiltrate within the deeper dermis and subcutis, with marked leukocytoclasia and hemorrhage. More superficially, the infiltrate showed lymphocytes and neutrophils, with lymphocytes being in intimate apposition to microvessels in the papillary and superficial half of the reticular dermis. The infiltrate is also permeative of the adventitial dermis of the eccrine coil. The PAS, GMS, and Gram stains were negative for microbial pathogens. The report was diagnostic for SS with subcutaneous involvement, compatible with a paraneoplastic neutrophilic dermatosis. The patient was started on a steroid taper (Figure 3). A bone marrow biopsy was performed, showing MDS with increased blasts and fibrosis. The patient was then started on induction chemotherapy with decitabine.

## Discussion

MDSs are clonal disorders of hematopoietic stem cells characterized by cytopenias, varying degrees of dysplasia, and a risk of progression to AML [8]. It is a disease of older adults, like our patient (72 years old), with approximately 86% of patients with MDS aged 60 years or older at the time of their diagnosis. The incidence varies from 120 to over 500 per million among older adults, with an annual prevalence of 60,000 to 120,000 in the U.S. population [9]. MDS is associated with environmental exposures, such as radiation and benzene [10]. MDS is a late toxicity of cancer treatment in individuals who have had a previous radiation treatment or radio-mimetic alkylating agent exposure [11], with a known latent period of five to seven years. Of note, our patient has a history of breast cancer treated with surgery (lumpectomy), chemotherapy, and radiation therapy 18 years ago. This history of radiation therapy could increase the risk of developing MDS.

Anemia dominates the early clinical presentation of MDS or may be part of a bi- or pancytopenia, of which patients may present with or without symptoms. Macrocytosis is expected, as well as the presence of large and agranular platelets. Our patient had bicytopenia at presentation, but this progressed to pancytopenia after two sessions of plasmapheresis when the WBC significantly dropped. Physical examination in MDS may be notable for signs of anemia (present in our patient) and splenomegaly (absent in our patient). About 20% of patients may have unusual skin findings, including SS (febrile neutrophilic dermatosis) [12,13], as seen in our patient.

The bone marrow is usually normal or hypercellular; however, it is found to be hypocellular in 20% of cases, for which differentiation from aplastic anemia will be required [14]. MDS (less than 20% blast cells in bone marrow) can progress to AML (20% or more blast cells in bone marrow). In this context, our patient had increased blasts in the range of 5%-10% and dysmegakaryopoiesis.

Prognostication is based on the International Prognostic Scoring System, involving the number and severity of cytopenias and cytologic abnormalities, with the opportunity for cure following hematopoietic stem cell transplantation [15]. Current management options include epigenetic modulators, which are thought to act through a demethylating mechanism. Hypomethylating agents, including azacitidine and decitabine, are frequently used [16]. Lenalidomide and human growth factors are frequently used to improve blood counts in addition to hemotransfusion. Our patient had remission following decitabine-based chemotherapy.

SS is a rare disease marked by the sudden onset of fever, leukocytosis, and erythematous plaques or nodules infiltrated by neutrophils [17]. Generally, SS presents as one of three clinical types: classic, cancer-associated, or drug-induced [18]. The distinct features of MASS are a) equal frequency in men and women, b) lack of preceding upper respiratory tract infection, and c) association with newly diagnosed or relapsed cancer [18]. Our patient was diagnosed with paraneoplastic SS (cancer-associated), specifically MDS-associated SS.

The most common symptom is fever, which may antedate the skin lesions. Skin lesions are painful, erythematous, and nodular or popular, and can coalesce and progress to form plaques [19]. MASS may uniquely present as bullous, ulcerated lesions with morphologic features of pyoderma gangrenosum. Cutaneous pathergy, such as in postmastectomy lymphedema, has also been observed to incite SS skin lesions. As many as 83% and 68% of patients have anemia and abnormal platelets, respectively, at presentation [19], similar to the findings in our patient.

There are no specific guidelines for treating MASS; therefore, it is managed just like classical SS by treating the underlying malignancy and with a course of systemic glucocorticoids at 1 mg/kg for three to four weeks and subsequently tapered over six weeks [20]. When treated early, fever, skin lesions, and other symptoms resolve within a few days. However, lower doses of corticosteroids are often needed for several weeks to months to prevent recurrence. Rarely, SS may persist for years. The use of topical, intralesional, and pulse steroids should be individualized. Second-line drugs include dapsone and cyclosporine, indomethacin, clofazimine, and chlorambucil [17,19]. Of note, our patient was initiated on oral prednisone at 1 mg/kg with subsequent tapering.

## Conclusions

Dermatologists, hematologists/oncologists, and primary physicians need to maintain a high index of suspicion for SS in patients diagnosed with a malignancy, especially those with a previous history of radiation therapy, who present with painful, erythematous nodules, papules, or plaques. A biopsy of skin lesions should be performed to confirm the diagnosis of SS and subsequently treated with steroids while treating the underlying malignancy. Based on the patient’s history, clinical course, imaging, and laboratory and pathology results, as well as response to therapy, our patient most likely had MASS in the setting of MDS. She underwent decitabine-based chemotherapy and a steroid course and is pending stem cell transplantation.

## References

[1] Zheng S, Li S, Tang S, Pan Y, Ding Y, Qiao J, Fang H (2020). Insights into the characteristics of Sweet syndrome in patients with and without hematologic malignancy. Front Med (Lausanne).

[2] Rochet NM, Chavan RN, Cappel MA, Wada DA, Gibson LE (2013). Sweet syndrome: clinical presentation, associations, and response to treatment in 77 patients. J Am Acad Dermatol.

[3] Ferea CR, Mihai SN, Balan G, Badescu MC, Tutunaru D, Tatu AL (2023). Sweet syndrome associated with myelodysplastic syndrome-a review of a multidisciplinary approach. Life (Basel).

[4] Vera-Lastra O, Olvera-Acevedo A, Pulido-Díaz N (2021). Transformation of a myelodysplastic syndrome to acute myeloid leukemia and concurrent necrotizing Sweet syndrome. Dermatol Reports.

[5] Gill HH, Leung AY, Trendell-Smith NJ, Yeung CK, Liang R (2010). Sweet syndrome due to myelodysplastic syndrome: possible therapeutic role of intravenous immunoglobulin in addition to standard treatment. Adv Hematol.

[6] Saygin C, Godley LA (2021). Genetics of myelodysplastic syndromes. Cancers (Basel).

[7] Toma A, Fenaux P, Dreyfus F, Cordonnier C (2012). Infections in myelodysplastic syndromes. Haematologica.

[8] Park M (2021). Myelodysplastic syndrome with genetic predisposition. Blood Res.

[9] Ma X (2012). Epidemiology of myelodysplastic syndromes. Am J Med.

[10] Schnatter AR, Glass DC, Tang G, Irons RD, Rushton L (2012). Myelodysplastic syndrome and benzene exposure among petroleum workers: an international pooled analysis. J Natl Cancer Inst.

[11] Sun LM, Lin CL, Lin MC, Liang JA, Kao CH (2015). Radiotherapy- and chemotherapy-induced myelodysplasia syndrome: a nationwide population-based nested case-control study. Medicine (Baltimore).

[12] Alderazi A, Rezigh AB (2023). An uncommon culprit of neutropenic fever: a case of Sweet syndrome following induction therapy for acute myeloid leukemia. Arch Clin Cases.

[13] Cook MR, Karp JE, Lai C (2022). The spectrum of genetic mutations in myelodysplastic syndrome: should we update prognostication?. EJHaem.

[14] Biswajit H, Pratim PP, Kumar ST, Shilpi S, Krishna GB, Aditi A (2012). Aplastic anemia: a common hematological abnormality among peripheral pancytopenia. N Am J Med Sci.

[15] Triantafyllidis I, Ciobanu A, Stanca O, Lupu AR (2012). Prognostic factors in myelodysplastic syndromes. Maedica (Bucur).

[16] Sorrentino VG, Thota S, Gonzalez EA, Rameshwar P, Chang VT, Etchegaray JP (2021). Hypomethylating chemotherapeutic agents as therapy for myelodysplastic syndromes and prevention of acute myeloid leukemia. Pharmaceuticals.

[17] von den Driesch P (1994). Sweet's syndrome (acute febrile neutrophilic dermatosis). J Am Acad Dermatol.

[18] Raza S, Kirkland RS, Patel AA, Shortridge JR, Freter C (2013). Insight into Sweet's syndrome and associated-malignancy: a review of the current literature. Int J Oncol.

[19] Cohen PR, Kurzrock R (1993). Sweet’s syndrome and cancer and cancer. Clin Dermatol.

[20] Cohen PR (2007). Sweet's syndrome--a comprehensive review of an acute febrile neutrophilic dermatosis. Orphanet J Rare Dis.

